# Probing variable range hopping lengths by magneto conductance in carbonized polymer nanofibers

**DOI:** 10.1038/s41598-018-23254-0

**Published:** 2018-03-21

**Authors:** Kyung Ho Kim, Samuel Lara-Avila, Hans He, Hojin Kang, Sung Ju Hong, Min Park, Johnas Eklöf, Kasper Moth-Poulsen, Satoshi Matsushita, Kazuo Akagi, Sergey Kubatkin, Yung Woo Park

**Affiliations:** 1 Department of Microtechnology and Nanoscience, Chalmers University of Technology, SE-412 96 Gothenburg, Sweden; 2 Department of Physics and Astronomy, Seoul National University, Seoul, 08826 Korea; 3 National Physical Laboratory, Hampton Road, Teddington, TW11 0LW UK; 4 Institut für Festkörperphysik, Leibniz Universität Hannover, Appelstraße 2, 30167 Hannover, Germany; 5 KIST Jeonbuk Institute of Advanced Composite Materials, Jeonbuk, 565-905 Korea; 6 Department of Chemistry and Chemical Engineering, Chalmers University of Technology, SE-412 96 Gothenburg, Sweden; 7 Department of Polymer Chemistry, Kyoto University, Katsura, Kyoto, 615-8510 Japan; 8 Institute of Applied Physics, Seoul National University, Seoul, 08826 Korea; 9 Department of Physics and Astronomy, University of Pennsylvania, Philadelphia, PA 19104 USA

## Abstract

Using magneto transport, we probe hopping length scales in the variable range hopping conduction of carbonized polyacetylene and polyaniline nanofibers. In contrast to pristine polyacetylene nanofibers that show vanishing magneto conductance at large electric fields, carbonized polymer nanofibers display a negative magneto conductance that decreases in magnitude but remains finite with respect to the electric field. We show that this behavior of magneto conductance is an indicator of the electric field and temperature dependence of hopping length in the gradual transition from the thermally activated to the activation-less electric field driven variable range hopping transport. This reveals magneto transport as a useful tool to probe hopping lengths in the non-linear hopping regime.

## Introduction

Variable range hopping (VRH) conduction has been investigated widely in non-crystalline and crystalline materials including amorphous carbons^1^, conducting polymers^2–4^, and semiconductor nanocrystals^5^. For disordered systems, Mott^6^ first pointed out that the hopping conductivity of the Miller-Abraham’s resistance network, taking into account both tunneling between localized wave functions and thermal activation, can be expressed as^7^,

1 $$\sigma \propto \exp [-\frac{2{r}_{hop}}{{\xi }_{0}}-\frac{{\rm{\Delta }}E}{{k}_{B}T}]$$

where *r*_*hop*_ is the temperature-dependent average hopping length, ${\xi }_{0}$ is the localization length at zero magnetic field, Δ*E* is the width of the energy interval near the Fermi level where hopping takes place, and *k*_*B*_*T* is the thermal energy. In the high temperature limit, transport is dominated by thermally activated nearest neighbor hopping, which results in Arrhenius-like conductivity. However, at lower temperatures, it might be energetically favorable for electrons to hop distances that in average are larger than the nearest-neighbor distance. In this conditions, the average hopping length follows a temperature dependence ${r}_{hop}\approx {\xi }_{0}({T}_{0}/T{)}^{s}$, resulting in the Mott’s law of VRH, where the conductivity is $\sigma (T)\propto \exp [-({T}_{0}/T{)}^{s}]$, with *s* = 1/(*d* + 1) for *d* dimensional systems, ${T}_{0}=\beta /({k}_{B}g({E}_{F}){{\xi }_{0}}^{d})$, *β* is a numerical constant and $g({E}_{F})$ is the density of state at the Fermi level^6,8^. In particular, for the Efros-Shklovskii systems (ES), where the Coulomb interaction is involved in the VRH conduction, the density of states at the Fermi level is modified by the formation of a soft Coulomb gap and the exponent *s* takes the constant value 1/2 regardless of dimensions^8–10^. Further lowering temperature, the conduction reaches to the activation-less regime, where the current-voltage (*I*-*V*) is temperature independent and highly non-linear^2,3,5,10–15^. In this low temperature regime, the average hopping length follows an electric field *F* dependence ${r}_{hop}\approx {\xi }_{0}({F}_{0}/F{)}^{s}$, resulting in the non-linear, activation-free, field driven transport $\sigma (F)\propto \exp [-({F}_{0}/F{)}^{s}]$ with ${F}_{0}$ a constant^2,3,5,10–14,16^. Yet, at the crossover between the thermally activated to the activation-less, electric field driven transport, the hopping length is a function of both the temperature and electric field, and thereby it cannot be determined solely from ${r}_{hop}\approx {\xi }_{0}({T}_{0}/T{)}^{s}$ nor from ${r}_{hop}\approx {\xi }_{0}({F}_{0}/F{)}^{s}$.

In this paper, we show that the effect of the magnetic field in transport can be used to probe the hopping lengths in ES-VRH systems in the crossover from the thermally activated to the activation-less electric field driven transport, and to quantify the relative effects of temperature and electric field to the average hopping distance. We focus on carbonized polyacetylene (CPA) and polyaniline (CPANI) nanofibers as ES-VRH model systems and investigate their magneto conductance (MC) up to *H* = 14 T as a function of bias voltage and temperature. The carbonized nanofibers become essentially amorphous carbon fibers after pyrolysis of polymer nanofibers at 800 °C^14,17–22^ and show characteristics of the ES-VRH conduction in the temperature and electric field dependence of conductivity^14^. These nano-materials are advantageous^2,5,14^ to study the crossover from thermal to electric field driven transport due to the possibility of applying very large electric fields in realistic experimental conditions. Additionally, because their polymer chain structure is modified during pyrolysis, polymer nanofibers serve as a test bed to explore the effects of polymer structural changes on MC. For PA and PANI nanofibers, pyrolysis at 800 °C results in dehydrogenation and cross-linking of adjacent polymer chains, yielding quasi amorphous, graphite-like carbon fibers^14,17–22^. In its pristine form, polyaniline (PANI) nanofibers display a finite and negative MC independent of the magnitude of the applied electric field. In sharp contrast, pristine polyacetylene (PA) nanofibers display a vanishing magnetoresistance (VMR) at high electric fields, which is attributed to charge being predominantly carried by spin-less topological solitons emerging from the peculiar backbone of PA (i.e. discontinuities – kinks, in the sequence of single and double bonds along the PA chain)^23–28^. Therefore, the VMR, (i.e. zero magnetoresistance at high electric fields) of PA nanofibers probes the spin-charge inversion of this material^23–28^, which is also important for exploring intrinsic conduction mechanism of conducting polymers^29–33^. In contrast to pristine PA nanofibers, both CPA and CPANI nanofibers display a negative MC that decreases in magnitude but remains finite with respect to the electric field. After carbonization of polymer chains, the non-trivial behavior of MC can be understood in the context of VRH, and we find that the electric-field and temperature dependence of MC can be used to probe the electric field and temperature dependence of hopping length. As it will be shown later, the weaker MC at high bias is not merely a consequence of Joule heating. Instead, we show that our measurements can be understood in the context of the wave function shrinkage model^9^, in which the negative MC is the result of magnetic confinement of localized states.

## Results and Discussion

**Figure 1:**
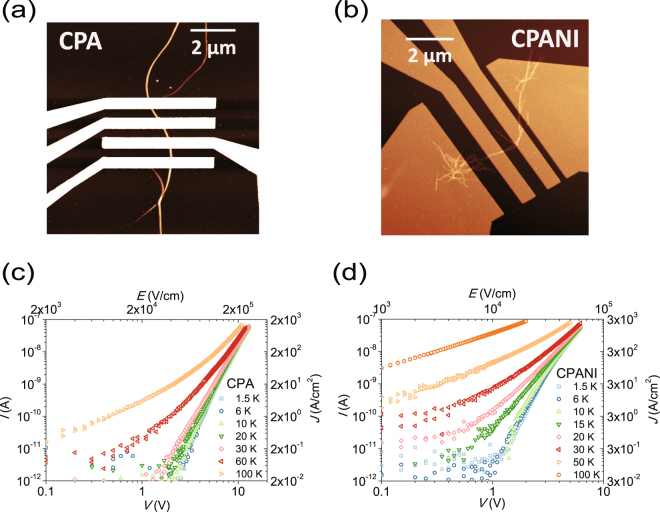
AFM images and *I*-*V* of CPA and CPANI nanofibers. AFM images of (**a**) CPA and (**b**) CPANI nanofibers with contact electrodes, respectively. *I*-*V* curves of (**c**) CPA and (**d**) CPANI nanofibers at 1.5 K ${\boldsymbol{\le }}\,$*T* ${\boldsymbol{\le }}\,$100 K in a double logarithmic scale.

Figure 1 shows the atomic force microscope (AFM) images of typical CPA and CPANI nanofibers, obtained by pyrolysis at 800 °C, as wells as the current voltage (*I-V*) characteristic at different temperatures of the CPA and CPANI nanofibers devices under study. The typical diameters (length) of the carbonized fibers are 15–80 nm (~10 μm), and their disordered graphite structure has been determined by Raman^14,17–20^ and XRD^17–20^ spectra. In the low temperature limit, for temperatures *T* < ~30 K (~10 K) for CPA (CPANI), the *I*-*V* characteristic follows the ES-VRH conduction for bias voltages above the transport gap *V* = ~2 V (~1 V) for CPA (CPANI), with $\sigma (F)\propto \exp [-({F}_{0}/F{)}^{1/2}]$ being essentially temperature independent^10–15^. For the high temperature limit, *T* > ~30 K (~10 K) for CPA (CPANI), the temperature dependence of transport properties is recovered due to the transition from the field-assisted ES-VRH to thermally activated VRH, with $\sigma (T)\propto \exp [-({T}_{0}/T{)}^{1/2}]$^8–15^. The disordered graphite structure of the carbonized nanofibers, determined by Raman spectra^14,17–20^ and XRD^17–20^, supports our interpretation of temperature dependence of currents because the amorphous structure allows the field driven hopping between localized states near Fermi level in a wide range of electric fields without breakdown.

**Figure 2:**
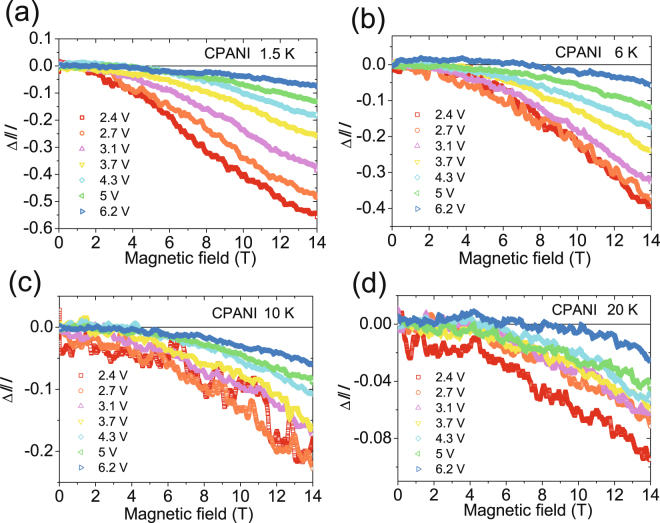
MC of the CPANI nanofiber at fixed voltages (${\boldsymbol{\Delta }}{\boldsymbol{I}}/{\boldsymbol{I}}$) at (**a**) 1.5 K, (**b**) 6 K, (**c**) 10 K, (**d**) 20 K. MC is systematically weaker at higher bias voltages and temperatures.

Figure 2(a–d) depict the transverse MC of the CPANI nanofibers (See also supporting info for MC of CPA), defined as MC = Δ*I*/*I* = [*I* (*H*) − *I* (0)]/*I* (0), with *I* the current through the device and *H* the applied perpendicular magnetic field. The MC of all our devices is finite and negative in the range of our measurements, with a parabolic magnetic field dependence visible at large magnetic fields. The MC has electric field dependence as well as temperature dependence: at fixed temperatures, the magnitude of MC decreases as the source-drain electric field increases and conversely, at a fixed bias, the magnitude of MC decreases as the temperature increases.

The decreasing magnitude of MC with increasing bias voltages has been reported frequently in low dimensional systems such as network of PANI nanofibers^4^, polymer nanofibers^24,25^, organic semiconductors^34^, and InSb nanowires^35^. This behavior has been interpreted within completely different theoretical frameworks depending on materials: for PANI nanofiber networks, decrease of activation energy at high electric field^4^; for polymer nanofibers, spin-less topological solitons associated with the electron-phonon interaction within the polymer chains^23–25^; for organic semiconductors, interactions between charged and uncharged species^34^; for InSb nanowires, breaking of spin-charge separation in Luttinger liquid in a magnetic field^35^.

**Figure 3:**
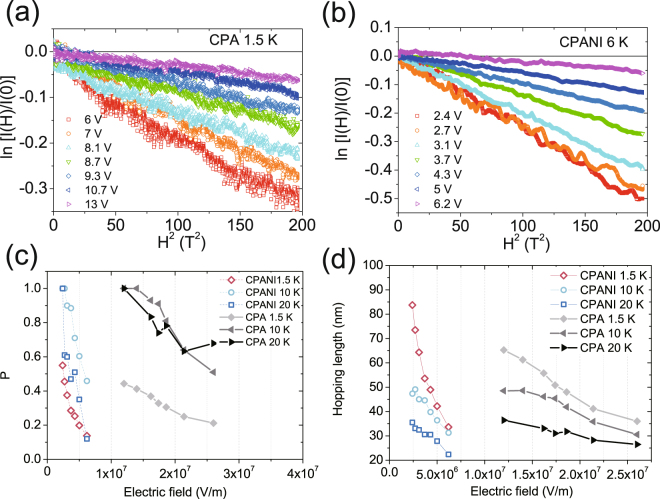
Analysis of MC in the context of Eq. 4 (**a**,**b**) ln[*I* (*H*)/*I* (0)] vs *H*^2^ plot of MC in (**a**) CPA at 1.5 K and (**b**) CPANI at 6 K. The linear dependence with decreasing magnitude of slopes at high electric field shows that the MC follows the wave function shrinkage model with electric field and temperature dependence of hopping length (Eq. 4). (**c**) Electric field and temperature dependence of the numerical variable *P*, which decreases as electric field increases. (**d**) The corresponding hopping lengths from Eq. 3.

For our devices, we propose a general explanation applicable to VRH systems that takes into account the effect of magnetic field and allows to probe length scales by MC in the non-linear *I*-*V* hopping regime as well. We consider the wave function shrinkage effect as the effect that explains the behavior of MC taking into account the ES-VRH conduction shown in these systems (Fig. 1)^14^. In this framework, the mechanism of MC is that the external magnetic field contracts localized wave functions in the direction perpendicular to the magnetic field, causing effectively longer hopping length and thereby reduces conductivity^9^. In the low magnetic field limit, when the localization length is smaller than the magnetic length, the MC of the ES-VRH is^9^:

2 $$\mathrm{ln}\,[I(H)/I(0)]\propto {{r}_{hop}}^{3}{\xi }_{0}/{\lambda }^{4}=k({r}_{hop},{\xi }_{0}){H}^{2},$$

where $\lambda =\sqrt{h/(2\pi eH})$ is the magnetic length. Figure 3(a,b) show the MC of the CPA and CPANI as a function of *H*^2^ as $\mathrm{ln}\,[I(H)/I(0)]$ vs *H*^2^ where the quadratic dependence is evident. The parabolic magnetic field dependence of Eq. 2 holds for magnetic field well below the critical field ${H}_{c}=6h/[2\pi e{{\xi }_{0}}^{2}{(T{}_{0}/T)}^{1/2}]$, which are 18 T (1.5 K), 46 T (10 K), and 58 T (20 K) for the CPA and 10.6 T (1.5 K), 21 T (6 K), 30 T (10 K), and 38 T (20 K) for the CPANI. These estimations explain the weak deviation in the MC of CPANI at 1.5 K in high magnetic fields^9,36^. The slope of the linear fit in Fig. 3(a,b), $k({r}_{hop},{\xi }_{0})$ at each voltage, is a function of both hopping length and the localization length, which qualitatively explains the electric field dependence of MC in the activation-less regime in the ES-VRH as ${r}_{hop}\approx {\xi }_{0}({F}_{0}/F{)}^{1/2}$. This relation ${r}_{hop}\approx {\xi }_{0}({F}_{0}/F{)}^{1/2}$ in the temperature independent high electric field, activation-less regime of the ES-VRH can be understood from the condition that the drop of the electric potential energy in a single hop is comparable with the width of energy where hopping takes place, $eF{r}_{hop}\approx {\rm{\Delta }}E$. Taking ${{r}_{hop}}^{-d}\approx N\propto {\int }_{{E}_{F}}^{{E}_{F}+\Delta E}g(E)dE\approx {\rm{\Delta }}{E}^{d}$ with the soft Coulomb gap in the density of states $g(E)\propto {|E-{E}_{F}|}^{d-1}$, and *d* the spatial dimension^[,5,8,9,11–13^ then electric field dependent hopping length, ${r}_{hop}\approx {\xi }_{0}({F}_{0}/F{)}^{1/2}$ gives rise to $\sigma (F)\propto \exp [-({F}_{0}/F{)}^{1/2}]$ in the activation-less regime given that $\sigma \propto \exp [-2{r}_{hop}/{\xi }_{0}]$^5,10–13^. At low electric fields and high temperatures where the current is temperature dependent, maximizing conductivity in Eq. 1 with the relation ${\rm{\Delta }}E\propto {{r}_{hop}}^{-1}$ results in ${r}_{hop}\approx {\xi }_{0}({T}_{0}/T{)}^{1/2}$ ^8–10^. In the moderate electric field regime where thermal activation and electric field driven contributions coexist, we expect that the MC is both electric field and temperature dependent. Therefore, we represent the hopping length phenomenologically as a function of temperature and electric field:

3 $${r}_{hop}\approx {\xi }_{0}[P({T}_{0}/T{)}^{1/2}+(1-P){({F}_{0}/F)}^{1/2}]$$

and rewrite Eq. 2 as^9^

4 $$\mathrm{ln}\,[I(H)/I(0)]=-t({{\xi }_{0}}^{4}/{\lambda }^{4}){[P{({T}_{0}/T)}^{1/2}+(1-P){({F}_{0}/F)}^{1/2}]}^{3}=k({\xi }_{0},P){H}^{2}\,(0\le P\le 1),$$

where *t* = 0.0015 is a numerical coefficient determined by the percolation method^9^ and *P* a phenomenologically introduced variable that changes gradually from 1 to 0 as the electric field increases; i.e. *P* = 1 in the low electric field Ohmic regime and *P* = 0 in the high electric field non-Ohmic regime. In this expression, *P* and 1-*P* are roughly the portion of the thermally activated and the electric field driven contributions, respectively.

We quantitatively analyze the MC of carbonized nanofibers in the context of Eq. 4. First we obtain localization lengths from $k({\xi }_{0},P=1)$ in Eq. 4 of MC at low voltages, 6 V (2.4 V) for CPA (CPANI), assuming the field-driven contribution is negligible (*P* = 1). The localization lengths in the temperature range 1.5 K ≤ *T* ≤ 20 K are found to be ${\xi }_{0}$ = 1.1 nm (1.5 K) − 1.9 nm (20 K) for the CPA nanofiber, using *T*_0_ = 7500 K, and *F*_0_ = 1.2 ${\rm{\times }}\,$10^9^ V/m from the temperature and electric field dependence of conductivity in the fit to $\sigma (T)\propto \exp [-({T}_{0}/T{)}^{1/2}]$ and $\sigma (F)\propto \exp [-({F}_{0}/F{)}^{1/2}]$, respectively^14^. In the CPANI nanofiber, the localization lengths in the range 1.5 K ≤ *T* ≤ 20 K are of the order of ${\xi }_{0}$ = 2.1 nm (1.5 K) − 2.9 nm (20 K), with *T*_0_ = 2900 K and *F*_0_ = 2.5 ${\rm{\times }}\,$10^8^ V/m^14^. The localization lengths except at 1.5 K were almost temperature independent, ${\xi }_{0}$ = 1.8−1.9 nm (2.8–2.9 nm) for CPA (CPANI), suggesting that *P* < 1 at 1.5 K at low voltages. The obtained values of *T*_0_ and localization length translate into higher density of states and easier thermal activation for CPANI compared to CPA. This is consistent with the lower resistance and the lower temperature threshold (~10 K) of the CPANI compared to that of CPA (~30 K) in Fig. 1 below which temperature independent *I*-*V* appears. We estimated *P* (Fig. 3(c)) in each electric field and temperature which gives the temperature independent localization length 1.8–1.9 nm and 2.8–2.9 nm for CPA and CPANI nanofibers, respectively, and calculated the average hopping length using Eq. 3 (Fig. 3(d)). Figure 3(c,d) shows the result that both *P* and the hopping length (Eq. 3) decrease as the electric field increases, demonstrating transition from thermally activated to activation-free field-driven transport as expected. Therefore, we conclude that the electric field and temperature dependence of MC arises from variation of the hopping length as a function of electric field and temperature.

Comparing the MC of pristine and carbonized PA nanofibers, the MC of carbonized polymer nanofibers decreases in magnitude but remains finite with respect to the electric field in contrast to pristine PA nanofibers, which show vanishing magnetoresistance at large electric fields. The difference of MC between pristine and carbonized PA nanofibers arises from the dissimilar material structure. In PA nanofibers, charge is transferred via spin-less defects in the double and single bonds of PA chains. Meanwhile, for carbonized nanofibers, charge is carried by hopping electrons and a vanishing MC in the activation-less, field driven regime would imply that the hopping length tends to zero, which is not plausible because the hopping length is limited by at least the nearest neighbor distance.

**Figure 4:**
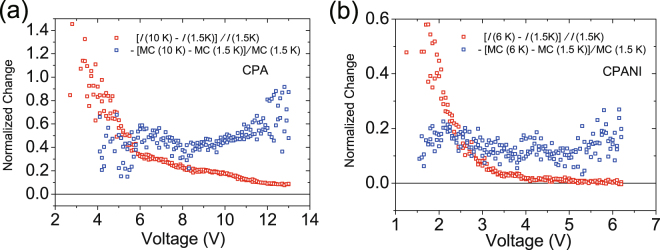
Comparison of voltage dependence between relative changes in current (Red) and MC at 14 T (Blue) [*I* (10 K) − *I* (1.5 K)]/*I* (1.5 K) (Red) of (**a**) CPA and (**b**) CPANI decrease monotonically as voltage increases. Contrarily, − [MC (10 K) −MC (1.5 K)]/MC (1.5 K) at *H* = 14 T (Blue) of (**a**) CPA and (**b**) CPANI are almost independent of voltages.

As alternative explanations to the observed MC in ES-VRH systems one might consider the spin dependent hopping model, in which the hopping length increases as a consequence of spin alignment in the presence of magnetic field^37^. However, this model predicts saturation of MC at high magnetic fields when all spins are aligned^37^, and this saturation has not been observed within the experimentally available magnetic fields. As a remark, trivial Joule heating is discarded as alternative explanation. If Joule heating was the major contribution to the weaker MC, the mechanism of temperature independent conductivity at higher bias must be also Joule heating. We compare bias dependence of current and that of MC at 14 T at two different temperatures. Figure 4(a) shows that the bias dependence of the normalized current difference between 10 K and 1.5 K, [*I* (10 K) – *I* (1.5 K)]/*I* (1.5 K), and that of the normalized change of MC at 14 T between 10 K and 1.5 K, – [MC (10 K) – MC (1.5 K)]/MC (1.5 K), of CPA is completely different. The former, [*I* (10 K) – *I* (1.5 K)]/*I* (1.5 K), decreases monotonically as a function of bias; However, the latter, – [MC (10 K) – MC (1.5 K)]/MC (1.5 K), is almost independent of bias. We repeat this for the CPANI nanofiber in Fig. 4(b), which shows that the behavior of the two is completely different as well. This is strong evidence that the MC as well as the temperature insensitive *I*-*V* at high bias are not due to Joule heating.

## Conclusions

In conclusion, we report temperature and electric field dependence of MC in carbonized polymer nanofibers and analyze it within the model of wave function shrinkage in the ES-VRH conduction. In contrast to the VMR of PA nanofibers, the MC of carbonized polymer nanofibers decreases but remains finite at high electric fields due to the profound change of chain structure after carbonization. The behavior of MC of carbonized polymer nanofibers is explained by the transition in the transport regimes, from thermally activated to field driven transport as the electric field increases, manifested by the variation of the hopping length as a function of electric field and temperature. We evaluate both the localization length and the hopping length and show that the hopping length is electric field dependent in the non-linear regime, resulting in the electric field dependence of MC. Therefore, the measurement of MC at different bias and temperatures in the non-linear VRH is a useful tool to probe the hopping length as a function of electric field and temperature.

## Methods

Synthesis, carbonization of PA and PANI nanofibers, and fabrication of the contacts are described in detail in our previous report^14^. In summary, an aligned PA film was synthesized by exposing acetylene gas of six-nine grade to an aligned nematic liquid crystal containing the Ziegler-Natta catalyst, Ti(O-n-Bu)4/AlEt3 by the gravity-flow method^38^. PANI nanofibers were synthesized by the rapid-mixing method using aqueous acidic solution of ammonium peroxydisulfate (0.8 mmol in 10 mL 1 N hydrochloric acid) as an oxidant and aqueous acidic solution of aniline (3.2 mmol in 10 mL of 1 N hydrochloric acid) and catalytic amount of *p*-phenylenediamine (5 mg) as a monomer and promoter for fiber growth, respectively^39^. The aligned PA film was dispersed in N,N-Dimethylformamide (DMF) with ultra-sonication and drop casted on 6 × 6 mm^2^ Si/SiO_2_ (300 nm) substrates and doped by gaseous iodine for one hour which prevents the PA film from decomposition during pyrolysis^18^. PANI nanofibers were dispersed in solution as synthesized and dropped on Si/SiO_2_ (300 nm). Pyrolysis of fibers for both PA and PANI nanofibers on Si/SiO_2_ (300 nm) substrates took place in a tube furnace at 800 °C for one hour under nitrogen flow with 1 °C/min of heating and cooling rate. Ti/Au (5/95 nm) contacts were defined by conventional e-beam lithography for electrical measurements. Magneto transport measurements were performed using an Oxford 14 T superconducting magnet with a Keithley 6517 electrometer in two-probe geometry. Temperature was controlled using a Neocera LTC-21 temperature controller together with a Variable Temperature Insert (VTI).

### Data availability

The datasets generated during and/or analysed during the current study are available from the corresponding author on reasonable request.

## Electronic supplementary material

Supplementary information
