# Supplementary material for: Probing variable range hopping lengths by magneto conductance in carbonized polymer nanofibers
Source: Sci Rep. 2018 Mar 21;8:4948. doi: 10.1038/s41598-018-23254-0 (PMC5862887; doi:10.1038/s41598-018-23254-0)
Supplement: Supplementary file 1 — Supplementary information [file 41598_2018_23254_MOESM1_ESM.pdf]

Supplementary Information of

Probing variable range hopping lengths by magneto conductance in carbonized polymer nanofibers

Kyung Ho Kim,[1,2*] Samuel Lara-Avila,[1,3] Hans He,[1] Hojin Kang,[2] Sung Ju Hong,[2,4] Min Park,[2,5] Johnas Eklöf,[6] Kasper Moth-Poulsen,[6] Satoshi Matsushita,[7] Kazuo Akagi,[7] Sergey Kubatkin,[1] and Yung Woo Park[2,8,9*]

[1]*Department of Microtechnology and Nanoscience, Chalmers University of Technology, SE-412 96 Gothenburg, Sweden*

[2]*Department of Physics and Astronomy, Seoul National University, Seoul, 08826, Korea*

[3]*National Physical Laboratory, Hampton Road, Teddington TW11 0LW, UK*

[4]*Institut für Festkörperphysik, Leibniz Universität Hannover, Appelstraße 2, 30167, Hannover, Germany*

[5]*KIST Jeonbuk Institute of Advanced Composite Materials, Jeonbuk 565-905, Korea*

[6]*Department of Chemistry and Chemical Engineering, Chalmers University of Technology, SE-412 96 Gothenburg, Sweden*

[7]*Department of Polymer Chemistry, Kyoto University, Katsura, Kyoto 615-8510, Japan*

[8]*Institute of Applied Physics, Seoul National University, Seoul 08826, Korea*

[9]*Department of Physics and Astronomy, University of Pennsylvania, Philadelphia, PA 19104, USA*

*Co-corresponding authors: kyungh@chalmers.se, ywpark@phya.snu.ac.kr

1. Magneto conductance of carbonized polyacetylene nanofibers

Figure S1 shows MC of a CPA nanofiber at different biases and temperatures. The behavior of MC in the CPA nanofiber is similar with that of the CPANI nanofiber in Fig. 2. The MC of CPA nanofibers are negative in the temperature range of 1.5 K $\leq T \leq$ 20 K and magnetic field up to H = 14 T. The magnetic field dependence is parabolic at low magnetic field limit. The MC of the CPA nanofiber has electric field dependence as well as temperature dependence: at fixed temperatures, the magnitude of MC decreases as the source-drain bias increases and conversely, at a fixed bias, the magnitude of MC decrease as the temperature increases.

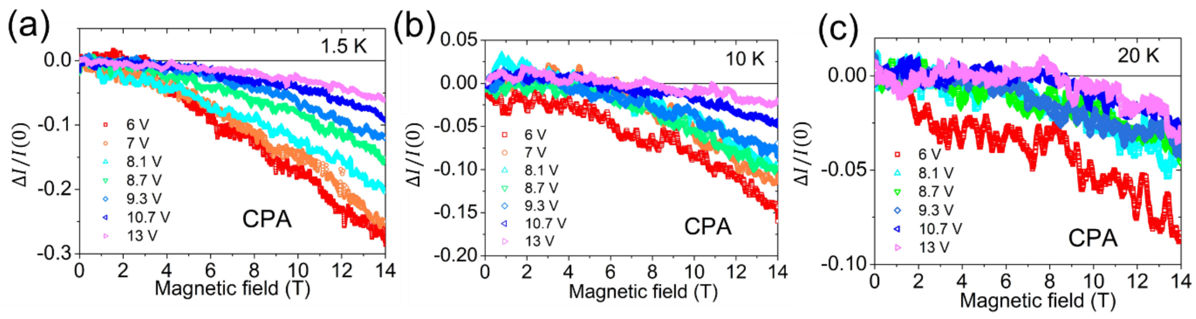

Figure S1. MC of the CPA nanofiber at fixed voltages ($\Delta I / I$) at (a) 1.5 K, (b) 10 K, and (c) 20 K. MC is systematically weaker at higher bias voltages and temperatures.

2. Electric field dependence of magneto conductance

Figure S2 shows continuous plot of bias dependence of MC at H = 14 T in the CPA and CPANI nanofibers. The MC (14 T) is calculated as [I (14 T) – I (0 T)]/I (0 T) from I–V measurements at 14 T and 0 T. Below and near transport gap, the MC is very noisy due to the small current level. The MC has clear and systematic electric field and temperature dependence, where at higher electric field and temperatures the MC becomes weaker. At 1.5 K, The MC tends to saturate to a finite value at high electric fields. This behavior might show that the hopping length saturates to the nearest-neighbor distance at high electric field limit.

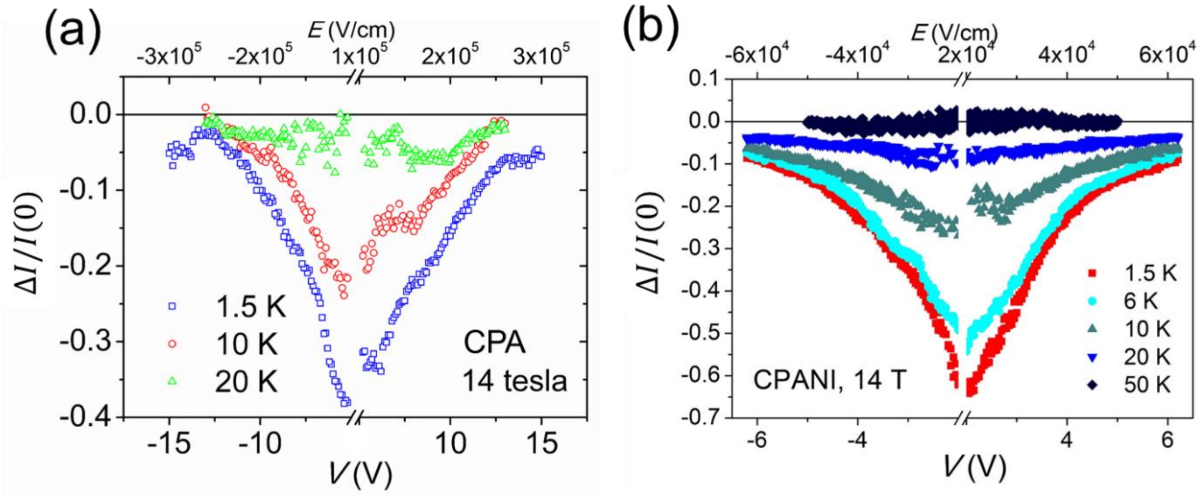

Figure S2. Electric field dependence of MC ($\Delta I / I$) for (a) CPA and (b) CPANI nanofibers. In this continuous electric field dependence, it is clear that the magnitude of MC decreases as a function of bias voltages. The MC is also weaker at higher temperatures.

3. Gate voltage dependence of transport in carbonized polymer nanofibers

We investigate back-gate voltage dependence of transport in carbonized polymer nanofibers fabricated on the Si/SiO_2 substrates. The thickness of SiO_2 dielectric is 300 nm and the Si back-gate is highly p-doped. The back-gate dependence of CPA nanofibers is not prominent as in Fig. S3(c). Contrarily, there is small (~10 % at 50 V) but apparent gate dependence in CPANI nanofibers (Fig. S3(f)).

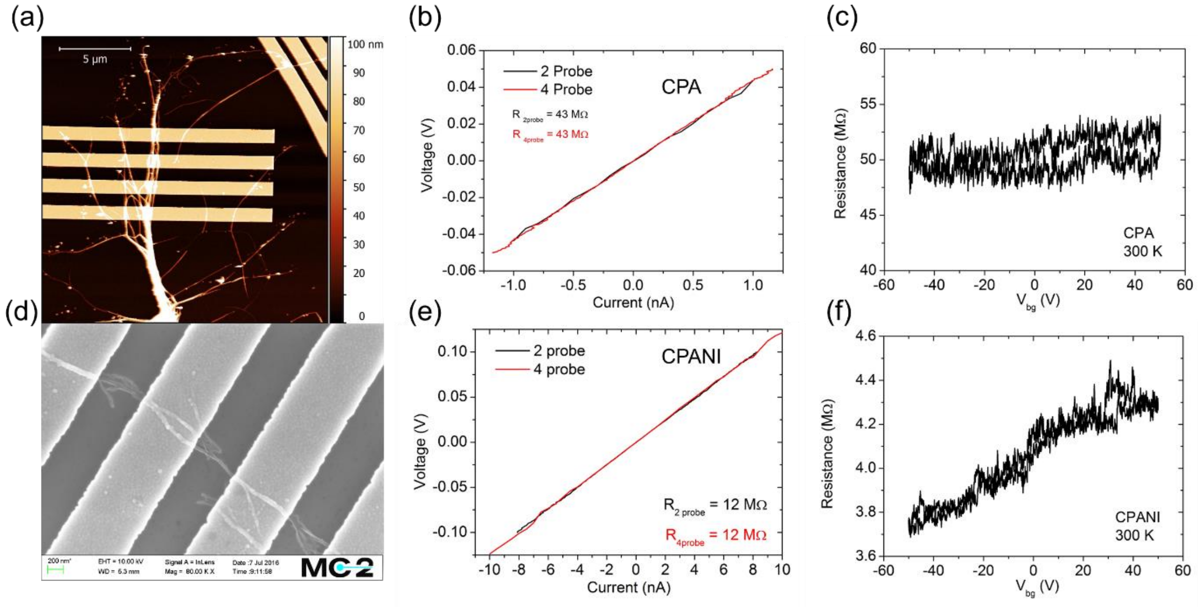

Figure S3. Back-gate dependence of transport at room temperature in CPA and CPANI nanofibers. (a,b,c) AFM image (a), *I*-*V* (b), and gate dependence of resistance (c) of a representative CPA nanofiber at room temperature. The scale bar in the AFM image is 5 μm. The two-probe and four-probe resistances are ~ 43 MΩ and the difference between the two (contact resistance) is negligible. The source-drain voltage during gate sweep was 100 mV. There is no clear gate dependence in CPA nanofibers. (d,e,f) SEM image (d), *I*-*V* (e), and gate dependence of resistance (f) of a representative CPANI nanofiber at room temperature. The scale bar of the SEM image is 200 nm. The two-probe and four-probe resistances are ~ 12 MΩ and the difference between the two (contact resistance) is negligible. The source-drain voltage during gate sweep was 50 mV. There is small gate dependence in CPANI nanofibers; the resistance increases in positive gate bias.

4. Comparison in diameter and conductivity between carbonized and pristine polymer nanofibers

We compare diameter and conductivity of 17 polyacetylene (PA), 33 CPA, 9 Polyaniline (PANI), and 15 CPANI nanofibers in Fig. S4. The conductivity of PA nanofibers is higher than that of CPA nanofibers and the conductivity of PANI nanofibers is similar with that of CPANI nanofibers. The diameter of all nanofibers are generally smaller than 100 nm. The decrease of diameter after carbonization is prominent in the comparison of diameter between PANI and CPANI nanofibers.

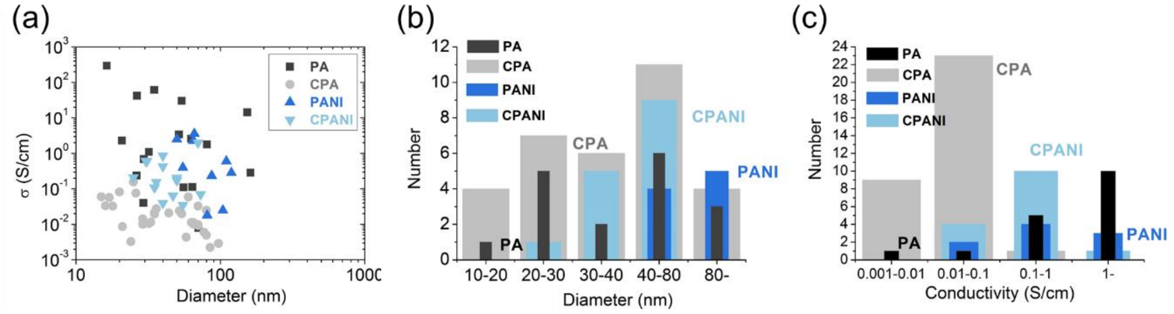

Figure S4. Statistics of 17 PA, 33 CPA, 9 PANI, 15 CPANI nanofibers. (a) As the diameter becomes smaller, the conductivity increases. Diameter of most of samples are smaller than 100 nm. (b,c) The distribution of diameter and conductivity of PA, CPA, PANI, and CPANI nanofibers

5. SEM and Raman spectra of the aligned polyacetylene film carbonized at 800 °C

Figure S5(a,b) shows SEM images of the aligned polyacetylene film[1] carbonized at 800 °C, where the carbonized polyacetylene (CPA) nanofibers are mutually assembled to form the bundles and the bundles are aligned parallel to the direction of the gravity flow. The film was doped by iodine in a glass vessel and carbonized at 800 ℃ using an electric furnace for 1 hour under flowing nitrogen gas. The hydrogen contents of the polyacetylene films carbonized at 800 °C are reported to be less than 1.0 wt %.[2,3] The theoretical elemental composition of polyacetylene is calculated for $[CH]_n$: C, 92.26; and H, 7.74. It is confirmed that the doped polyacetylene is almost completely carbonized at the heating temperature of 800 °C. Figure S6(a,b) shows Raman-scattering spectrum of the aligned polyacetylene film carbonized at 800 °C. The Raman spectrum showed broad disorder (D) and graphite (G) bands at 1346 cm^{-1} and 1585 cm^{-1}, respectively. A Raman intensity ratio of the G- and D-bands (I_G/I_D) was determined to be 1.03 from the Raman spectrum. This result indicates that the carbonized polyacetylene film exists in an almost amorphous state.

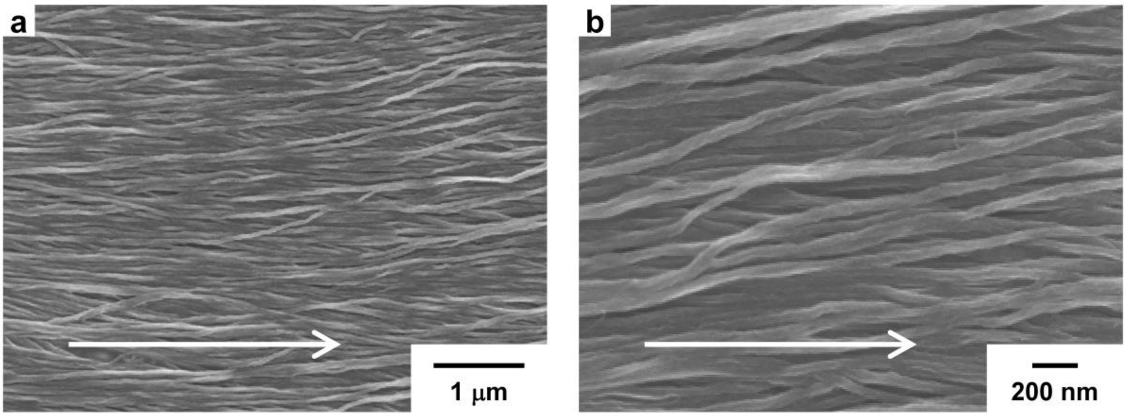

Figure S5. SEM images of the aligned polyacetylene film carbonized at 800 °C. The arrows indicate the direction of the gravity flow.

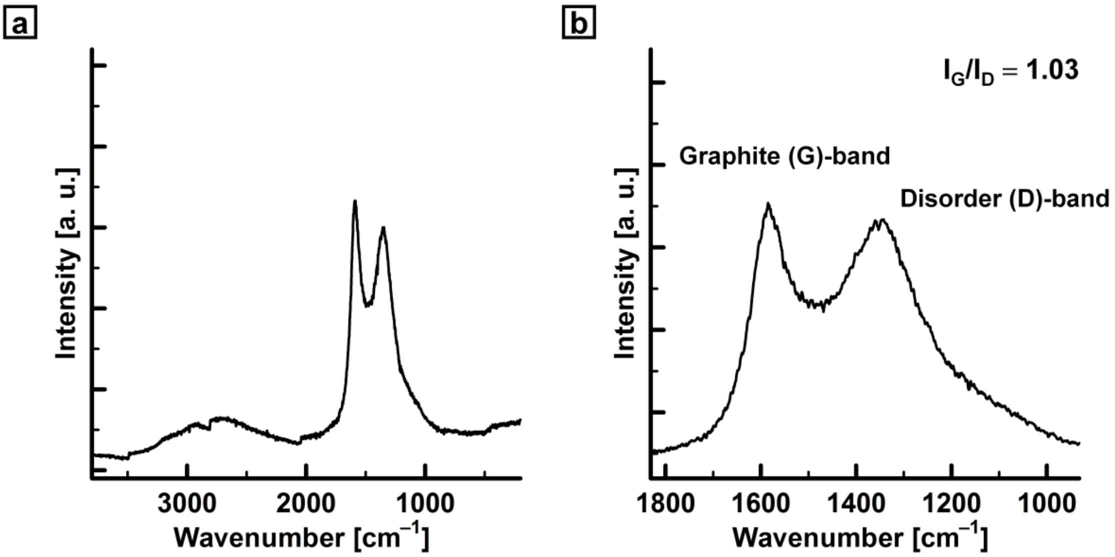

Figure S6. Raman spectrum of carbonized polyacetylene film in Fig.S5 (a) Raman-scattering spectrum of the aligned polyacetylene film carbonized at 800 °C and (b) its magnified spectrum.

References

1. Kyotani, M., Matsushita, S., Goh, M., Nagai, T., Matsui, Y. & Akagi, K. Entanglement-free fibrils of aligned polyacetylene films that produce single nanofibers. *Nanoscale* **2**, 509–514 (2010).

2. Matsushita, S., Kyotani, M. & Akagi, K. Hierarchically controlled helical graphite films prepared from iodine-doped helical polyacetylene films using morphology-retaining carbonization. *J. Am. Chem. Soc.* **133**, 17977–17992 (2011).

3. Matsushita, S. & Akagi, K. Macroscopically aligned graphite films prepared from iodine-doped stretchable polyacetylene films using morphology-retaining carbonization. *J. Am. Chem. Soc.* **137**, 9077–9087 (2015).
